# Assessment of various rotary file systems with different taper for canal cleaning: An *in vitro* study

**DOI:** 10.6026/973206300211486

**Published:** 2025-06-30

**Authors:** Shrishtee Priya, Gongalareddy Sai Vineetha, Deepika Yadav, Subasish Behera, Bibyaswan Chakrabarti, Soumyaranjan Nanda, Megha Patel

**Affiliations:** 1 Department of Conservative Dentistry and Endodontics, Vananchal Dental College, Garhwa, Jharkhand, India; 2 G. Pullareddy Dental College and Hospital, Kurnool, Andhra Pradesh, India; 3 Consultant Endodontist, Captain Nandlal Yadav Multi-Speciality Hospital, Rewari, Haryana, India; 4 Department of Conservative Dentistry and Endodontics, Government Dental College and Hospital (SCB), Manglabag, Cuttack, Odisha, India; 5 Department of Pediatric & Preventive Dentistry, College of Dental Science and Hospital, Rau, Indore, India; 6 Endodontics, Private Practitioner, Cuttack, Odisha, India; 7 Department of Pediatric and Preventive Dentistry, Karnavati School of Dentistry, Karnavati University, Gandhinagar, Gujarat, India

**Keywords:** Rotary file systems, taper, canal cleaning, debris removal, HyFlex EDM, protaper gold, trunatomy, endodontics

## Abstract

A key part of performing successful root canal therapy is effective root debridement. Hence, mandibular premolars were used to compare how well ProTaper Gold, HyFlex EDM and TruNatomy rotary file systems remove debris during cleaning. We instrumented 30 teeth and examined them through a stereomicroscope to see how much residual debris was left after canal shaping. The HyFlex EDM system was much better at cleaning, especially in the apical third (p < 0.05). Thus, we show that a good taper in the design of the file helps clean the canal better.

## Background:

Careful removal of infected tissues in a root canal is very important for achieving good endodontic treatment results. Root canal instrumentation aims to remove dead tissue, bacteria and pieces of tooth, allowing the canal to be thoroughly cleaned and sealed [1]. Obtaining this result is assisted by adding support from both mechanical instrumentation and chemical irrigation. Nonetheless, the changes in a canal's structure present many obstacles to getting the canal completely free of bacteria and debris, most especially in the apical third [2]. The use of nickel-titanium rotary instruments has greatly boosted the reliability and security in root canal therapy. NiTi rotary systems have greater adaptability, decrease the risk of errors and produce more certain results, compared to traditional stainless-steel hand files [3]. Many experts believe the taper is a top design feature for rotary instruments as it influences their cleansing, shaping skills and how well they remove debris [4]. Taper is about making the file gradually thicker by 1 mm for each millimeter along its cutting area and the rate of change can be steady, increasing, or decreasing. There are a number of rotary systems on the market today, all with special tapers and improvements to their metal to increase their performance. The ProTaper Gold uses a progressively shaped design and a special alloy, giving it both added flexibility and strength against fatigue [5]. The files produced by HyFlex EDM contain variable taper and adaptive shape memory thanks to electrical discharge machining technology, making them better at maintaining the location of the canal and cleaning away debris [6]. TruNatomy files are built to have a continuous, thin taper to keep as much dentin as possible during conservative canal shaping [7]. While a lot has been achieved, there is still no agreement that one taper is better for cleaning than another when used in different areas of the canal. Some experts report that a quicker taper improves how well irrigants clean the canal and remove debris, although others argue that selecting a slower taper allows you to preserve the outer cervical part of the tooth and keeps debris from moving into the canal [8, 9]. Therefore, carefully comparing rotary systems with various tapered designs can inform guidance for clinical decisions. The objective of this *in vitro* study is to evaluate and compare the cleaning efficacy of three NiTi rotary file systems-ProTaper Gold, HyFlex EDM and TruNatomy-each distinct in its taper configuration, on extracted human premolar teeth. Tyherefore, it is of interest to assess the removal of debris from the coronal, middle and apical thirds, thereby delivering a deeper understanding of the role of taper design in the efficient cleaning of root canals.

## Materials and Methods:

This *in vitro* experimental study was conducted on thirty freshly extracted, intact, human mandibular premolars with single, straight canals. Teeth exhibiting caries, fractures, calcifications, or multiple canals were excluded. All selected teeth were thoroughly cleaned of soft tissue and calculus using an ultrasonic scaler and stored in 0.1% thymol solution until use to prevent microbial growth.

## Sample preparation:

Standard access cavities were prepared using a high-speed diamond round bur under water coolant. A size 10 K-file was introduced into the canal until its tip was visible at the apical foramen and the working length was established by subtracting 1 mm from this measurement. Canals were initially instrumented with a size 15 K-file to ensure patency.

## Grouping of samples:

The specimens were randomly allocated into three groups (n = 10 per group) based on the rotary system used:

[1] Group I - ProTaper Gold (Dentsply Sirona): progressive taper

[2] Group II - HyFlex EDM (Coltene): variable taper

[3] Group III - TruNatomy (Dentsply Sirona): constant taper

Instrumentation in each group was carried out using a torque-controlled endodontic motor as per the manufacturer's recommendations. The canals were irrigated during instrumentation with 3 mL of 3% sodium hypochlorite after each instrument change and finally flushed with 5 mL of 17% EDTA, followed by 5 mL of distilled water to remove the smear layer and residual chemicals.

## Debris evaluation:

Following instrumentation, the roots were longitudinally grooved with a diamond disc and split into two halves using a chisel. The canal walls of each half were examined under a stereomicroscope at 10x magnification. Photographic images of the coronal, middle and apical thirds were captured and scored for debris using a modified 5-point scale:

[1] Score 1: Clean canal wall, few debris particles

[2] Score 2: Minor amount of debris

[3] Score 3: Moderate debris covering less than half the canal wall

[4] Score 4: Considerable debris covering more than half the wall

[5] Score 5: Heavy debris covering the entire canal wall

Two independent observers evaluated the specimens blindly. In case of disagreement, a consensus was reached after joint review.

## Statistical analysis:

The mean debris scores for each group and canals third were calculated. Intergroup comparisons were done using one-way analysis of variance (ANOVA) followed by Tukey's post hoc test for pairwise comparisons. A p-value of less than 0.05 was considered statistically significant. Statistical analysis was performed using SPSS software version 25.0 (IBM Corp., Armonk, NY, USA).

## Results:

All 30 samples were successfully instrumented without file separation or canal blockage. The cleanliness of canal walls was assessed in the coronal, middle and apical thirds and debris scores were assigned using the predefined 5-point scoring system. The mean debris scores and standard deviations for each group are presented in Table 1 (see PDF). As seen in Table 1 (see PDF), the HyFlex EDM group demonstrated the lowest mean debris scores across all thirds of the canal, particularly in the apical third, where the score was significantly lower (1.6 ± 0.5) compared to ProTaper Gold (2.5 ± 0.6) and TruNatomy (2.8 ± 0.7). The TruNatomy group consistently showed higher debris scores, indicating reduced cleaning efficiency. Statistical analysis using one-way ANOVA revealed a statistically significant difference in debris scores among the three groups in the apical third (p = 0.012), while the differences in the coronal and middle thirds were not statistically significant (p> 0.05). Pairwise comparisons using Tukey's test indicated that HyFlex EDM was significantly more effective than TruNatomy in apical cleaning (p< 0.05) (Table 2 - see PDF). In summary, HyFlex EDM outperformed the other two systems in cleaning efficacy, especially in the critical apical third. This suggests that variable taper design may contribute to enhanced debridement in anatomically challenging areas of the canal.

## Discussion:

The primary objective of root canal instrumentation is to eliminate all remnants of necrotic pulp, debris and microbes. It is crucial to implement this procedure to guarantee that the disinfection is thorough and that the likelihood of reinfection in the upper portion of the teeth is minimised, as cleaning this area can be exceedingly intricate [1, 2]. The effects of rotary NiTi files, including ProTaper Gold, HyFlex EDM and TruNatomy, in clearing root canal debris were examined during this study, focusing on how taper affects results. Results from our study showed that HyFlex EDM, with a variable taper, cleaned the canal best in the apical area. These results support previous studies, which suggest that adaptive alloys and variable taper structures make it easier to center the canal and minimize debris [3, 4]. The EDM process used to make HyFlex files increases both their resistance to breakage and their flexibility, helping them better follow curved canal parts, especially in difficult narrow spots near the bottom [5]. However, TruNatomy with a narrow, steady taper did not clean well, especially near the tip, in all the thirds tested. Despite trying to save pericervical dentin and avoid widening the canals, the small taper of TruNatomy could impede both irrigant flow and disruption of the debris at the base. This is consistent with the results of Peters *et al.* and Plotino *et al.* which demonstrate that large taper sizes typically facilitate the cleaning and flushing of crowded tooth areas [8, 9].

ProTaper Gold was able to remove debris from the coronal and middle thirds somewhat well. With its gradual tip, the instrument can enlarge the canal well in these areas, though stiffness at the end could make it harder to shape the tip [10]. Previous findings point to the possibility that avulsion of the root with instruments that are tapered at the coronal end may result in less debris being removed from the apical third [11, 12]. Although rotary tools have improved, removing all debris from a canal is still difficult, mainly because of isthmuses, fins and lateral canals [13]. Additional irrigation methods, such as passive ultrasonic irrigation and apical negative pressure, are required due to the fact that debris may persist in the canal following standard cleaning procedures, as indicated by research [14, 15]. Although we did not use these models in our study, adding them to future research could give more insights into taper cleaning performance. Here, review of the canals using a stereomicroscope showed the relative distribution of debris near the canal orifices. Even though this method was confirmed by former studies, factors like the possibility of scoring errors and biased opinions still affect it [16, 17]. To remove bias, we made sure all evaluators worked blindly and that we discussed and resolved inconsistencies in place. Clinicians will be able to determine the optimal file system for each patient based on the findings of this study. If the situation requires cleaning the central part of the root, such as when periapical involvement is involved, a system featuring variable taper and greater apical efficiency, such as HyFlex EDM, may be better suited. On the other hand, if little of the dentin can be removed, TruNatomy may be recommended, along with using certain irrigation steps to further clean.

## Conclusion:

Cleaning apical canals was more effective with HyFlex EDM than with ProTaper Gold and TruNatomy. Further, tapered form helps increase the cleaning action by giving the canal better adaptability and flexibility. How a canal is prepared depends greatly on the type of taper applied.
